# Expression and purification of the antimicrobial peptide GSL1 in bacteria for raising antibodies

**DOI:** 10.1186/1756-0500-7-777

**Published:** 2014-11-04

**Authors:** Sathiyamoorthy Meiyalaghan, Julie M Latimer, Andrew V Kralicek, Martin L Shaw, John G Lewis, Anthony J Conner, Philippa J Barrell

**Affiliations:** The New Zealand Institute for Plant & Food Research Ltd, Private Bag 4704, Christchurch, New Zealand; The New Zealand Institute for Plant & Food Research Ltd, Private Bag 92169, Auckland, New Zealand; Steroid & Immunobiochemistry Laboratory, Canterbury Health Laboratories, P.O. Box 151, Christchurch, New Zealand; AgResearch Limited, Grasslands Research Centre, Tennent Drive, Private Bag 11008, Palmerston North, New Zealand

**Keywords:** Snakin/GSL peptides, Antimicrobial, Thioredoxin, wheat-germ expression, Antibodies

## Abstract

**Background:**

The Gibberellin Stimulated-Like (GSL) or Snakin peptides from higher plants are cysteine-rich, with broad spectrum activity against a range of bacterial and fungal pathogens. To detect GSL peptides in applications such as western blot analysis and enzyme-linked immunosorbent assays (ELISA), specific antibodies that recognise GSL peptides are required. However, the intrinsic antimicrobial activity of these peptides is likely to prevent their expression alone in bacterial or yeast expression systems for subsequent antibody production in animal hosts.

**Results:**

To overcome this issue we developed an *Escherichia coli* expression strategy based on the expression of the GSL1 peptide as a His-tagged thioredoxin fusion protein. The DNA sequence for the mature GSL1 peptide from potato (*Solanum tuberosum* L.) was cloned into the pET-32a expression vector to produce a construct encoding N-terminally tagged his_6_-thioredoxin-GSL1. The fusion protein was overexpressed in *E. coli* to produce soluble non-toxic protein. The GSL1 fusion protein could be easily purified by using affinity chromatography to yield ~1.3 mg of his_6_-thioredoxin-GSL1 per L of culture. The fusion protein was then injected into rabbits for antibody production. Western blot analysis showed that the antibodies obtained from rabbit sera specifically recognised the GSL1 peptide that had been expressed in a wheat germ cell-free expression system.

**Conclusion:**

We present here the first report of a GSL1 peptide expressed as a fusion protein with thioredoxin that has resulted in milligram quantities of soluble protein to be produced. We have also demonstrated that a wheat germ system can be used to successfully express small quantities of GSL1 peptide useful as positive control in western blot analysis. To our knowledge this is the first report of antibodies being produced against GSL1 peptide. The antibodies will be useful for analysis of GSL1peptides in western blot, localization by immunohistochemistry (IHC) and quantitation by ELISA.

## Background

**Figure 1:**
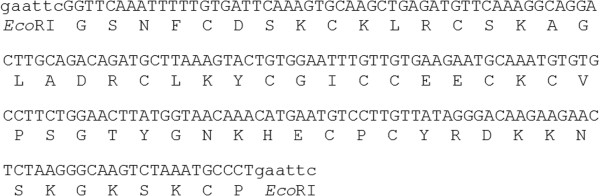
**The DNA and amino acid sequence of the mature GSL1 (Genbank accession FJ195646) from** ***Solanum tuberosum*** **, after cleavage of the N-terminal signal sequence.** Restriction endonuclease recognition sites for *Eco*RI added for cloning purposes are indicated in lower case letters.

Small antimicrobial peptides are popular targets for engineering plants in order to confer resistance to a range of microbial diseases [1–5]. The Gibberellin Stimulated-Like (GSL) peptides GSL1 and GSL2 (also known as Snakin-1 and Snakin-2 [6]) are one such group. They are cysteine-rich peptides from potato (*Solanum tuberosum* L.) (Figure 1) that have been shown to have antimicrobial activity against a wide range of bacteria and fungi [7–11], as well as nematodes [12]. GSL peptides are also considered to be important in plant developmental processes such as cell division, and stress responses regulating redox homeostasis [13, 14]. This is supported by the failure to recover viable plants following potato transformation with antisense constructs of *GSL* genes [6]. In contrast, over expression of *GSL* genes in potato does not cause obvious changes in plant phenotype [15]. GSL peptides have a very similar spectrum of activity against microbes [8, 9]. They induce rapid aggregation of both Gram-negative and Gram-positive bacteria, and although this response does not correlate directly with antimicrobial activity, it may play an *in vivo* role in controlling pathogen migration [7, 9, 11]. Transgenic plants over-expressing *GSL* genes have been shown to have increased resistance to a range of microbial pathogens [3, 15–17].

GSL1 is translated with a leader sequence, which is processed to yield a mature protein [8, 9] (Figure 1). To determine a protein’s expression pattern by western blot, localization by immunohistochemistry (IHC) and quantitation by ELISA, antibodies that selectively identify the protein of interest are required. Antibodies are routinely generated by injection of the protein (antigen) into a host animal such as rabbit or mouse. After a number of weeks, sera obtained from the host animal can then be tested for the presence of antibodies that selectively recognise the antigen. To elicit an immune response in the host animal, it is generally desirable to inject milligram quantities of the antigen. *Escherichia coli*–based bacterial expressions systems are commonly used to generate such recombinant proteins [18].

The intrinsic antimicrobial activity of antimicrobial peptides may prevent their expression in *E. coli* [19]. However, several proteins and peptides have been shown to be successful as fusion partners with antimicrobial peptides to enable their expression in *E. coli* [20–22]. The fusion protein is thought to protect the peptide from proteolytic cleavage and block the peptide’s antimicrobial activity against the expression host [23]. GSL1 has previously been expressed in *E. coli* by N-terminally tagging the peptide with the 22 amino acid *pelB* leader sequence enabling secretion into the bacterial periplasmic space [11]. However, to enable antibody production it is recommended that small peptides be coupled to a carrier protein to elicit a good immune response [24]. We have previously shown the utility of using thioredoxin as a fusion partner with an antimicrobial peptide to produce recombinant protein in *E. coli* for antibody production [25]. We chose the expression vector pET-32a to generate N-terminally tagged his_6_-thioredoxin-mature GSL1 fusion protein. The recombinant fusion protein was non-toxic to the host bacterium *E. coli* and protein was recovered in a soluble form. Sufficient recombinant GSL1 fusion protein was isolated and purified from *E. coli* in soluble form for injection into rabbits. Antibodies were obtained from rabbit sera that selectively recognised synthetic GSL1 in western blot analysis of GSL1 peptide produced in a wheat germ cell-free expression system. Our work is the first report on the successful soluble expression of recombinant GSL1 fusion protein and the generation of anti-GSL1 antibodies.

## Results

### Overexpression of the his_6_-thioredoxin-GSL1 fusion protein in *E. coli*

**Figure 2:**
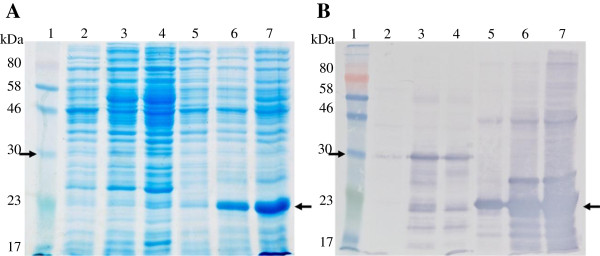
**SDS-PAGE and western blot analysis of N-terminally tagged his**_**6**_ **-thioredoxin GSL1 peptide expressed in** ***E. coli.*** **A**. SDS-PAGE analysis of the induced expression of his_6_-thioredoxin-GSL1 (Lanes 2–4 are 0, 2 and 4 hours) , and his_6_-thioredoxin tag only (Lanes 5-7 are 0, 2 and 4 hours). The expected size for his_6_-thioredoxin alone was approximately 23 kDA, and 30 kDa for N-terminally tagged his_6_-thioredoxin GSL1. Lane 1 contains molecular weight standards. An arrow indicates each expressed protein band. **B**. Western blot analysis of the same fractions using an anti-thioredoxin antibody.

The his_6_-thioredoxin-GSL1 fusion protein was overexpressed in the *E. coli* strain BL21 (DE3) using the pET-32a vector. In comparison with thioredoxin alone (Figure 2A, lanes 5-7, right arrow), very little GSL1–thioredoxin fusion protein was expressed (Figure 2A, lanes 2–4), as judged by Coomassie staining (Figure 2A, left arrow). However, western blot analysis using anti-thioredoxin antibodies showed that fusion protein of expected molecular weight of approximately 27 kDa was expressed for pET-32a+GSL1 (Figure 2B, left arrow, lanes 2–4). Maximum production of the fusion protein was achieved within 2 h (Figure 2B lane 3), without any apparent toxicity to the bacteria. Sufficient GSL1 fusion protein was judged to be produced in the soluble fraction after cell lysis to proceed with purification on a larger scale.

### Purification of his_6_-thioredoxin-GSL1 fusion protein and generation of antibodies in rabbits

**Figure 3:**
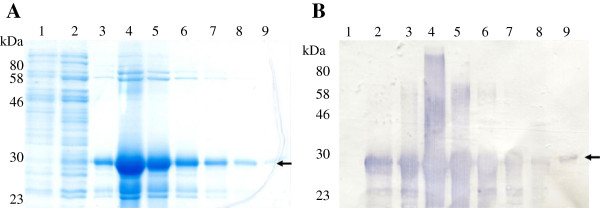
**SDS-PAGE and western blot analysis of protein fractions from the IMAC purification of his**_**6**_ **-thioredoxin-GSL1. A**: SDS-PAGE analysis of the fractions obtained. Lane 1: flow through from column; lane 2: eluate after first wash; lanes 3-9, fractions after elution buffer was applied. The arrow indicates the GSL1 fusion protein. **B**: Western blot analysis of the same protein fractions using an anti-thioredoxin antibody.

We successfully employed an IMAC purification process utilising the metal-binding polyhistidine (His.Tag) present as part of the thioredoxin fusion protein. The GSL1 fusion protein was purified to high purity. Figure 3A shows SDS-PAGE analysis by Coomassie staining of purified GSL1 fusion protein through the IMAC column purification process. Lane 1 shows protein fractions unbound by the column (flow through); Lane 2 shows the eluate after the first wash showing many non-specific protein bands; and Lanes 3-9 show the fractions after elution buffer was applied. The arrow indicates the GSL1 fusion protein, with the majority of the fusion protein eluting in fractions 5 and 6. Western blot analysis using an anti-thioredoxin antibody confirmed the successful purification of protein after elution from the column (Figure 3B, arrow). It was estimated that approximately 1.3 mg of purified concentrated fusion protein was obtained per litre of bacterial culture. The soluble fusion protein obtained was used for injection into two rabbits. Three sub-cutaneous injections of approximately 0.3 mg of fusion protein in a volume of 500 μL per injection were made four weeks apart. Total sera were collected two weeks after the final injection.

### Wheat germ cell-free expression of his_6_-GSL1 to confirm potency of anti-GSL antibody sera

In order to confirm the specificity of the anti-GSL1 antibodies, GSL1 peptide was required as positive controls. We purchased commercially synthesised mature GSL1 peptide to use as positive controls in our experiments. We attempted to solubilise the peptide in water; acetic acid; SDS loading buffer; SDS loading buffer +2- mercaptoethanol; isopropanol; methanol; ethanol; acetonitrile; trifluoroactic acid; and DMSO (data not shown). However, it was not possible to solubilise the supplied lyophilised peptide. Instead we cloned the DNA sequences encoding the mature GSL1 peptide into a wheat germ cell-free expression vector. The *GSL1* cDNA sequence was cloned into the pEU-DEST vector [26]. Expression of the *GSL1* construct resulted in translated GSL1 protein, as judged by western blot analysis using poly-His antibodies (data not shown).

**Figure 4:**
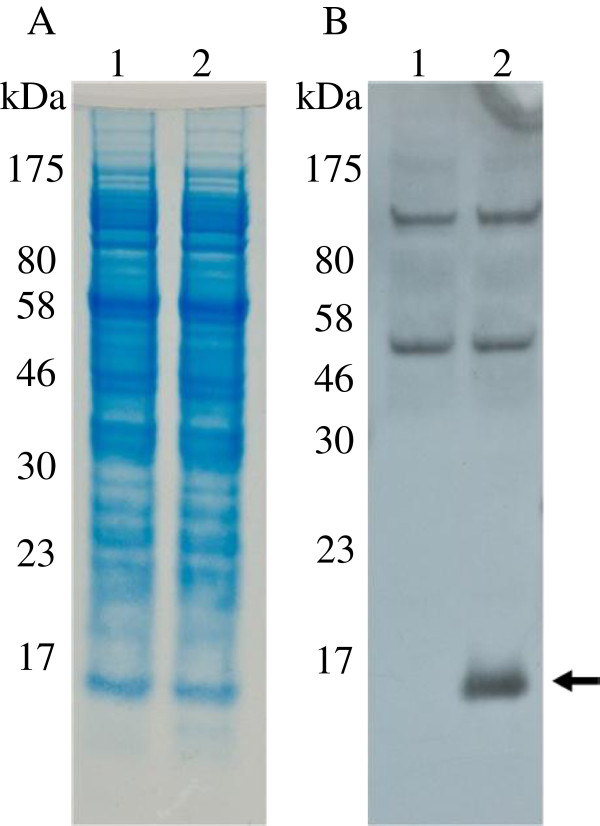
**SDS-PAGE and western blot analysis of wheat germ cell-free expressed GSL1. A**: Coomassie stained fractions. **B**: Western blot analysis using total sera raised against the GSL1 fusion protein. Lane 1: no expression control; Lane 2: his_6_-GSL1. The arrow in panel B indicates the GSL1 peptide.

Figure 4 shows Coomassie staining (A) and western blot analysis (B) of total protein from the wheat germ cell-free system. The vector control is shown in Lane 1, with Lane 2 containing the GSL1 protein. Western blot analysis was performed with total sera obtained from a rabbit injected with the GSL1 fusion protein. Using a dilution of the resulting total sera of 1:400 and 1:100,000 goat anti-rabbit conjugated to horseradish peroxidase, developed using peroxide and luminol/enhancer, antibodies successfully recognised the synthesised GSL1 (Figure 4B, arrow). The western blot shows that the sera obtained contained selective antibodies against the GSL1 peptide.

## Discussion

We have successfully demonstrated that the antimicrobial GSL1 peptide can be expressed as a thioredoxin fusion protein in a bacterial system to produce milligram quantities of soluble protein (Figures 2 and 3). A potato SN1 (GSL1) protein was previously expressed in *E. coli* strain BL21 (DE3) using the *pelB* leader sequence to facilitate localization to the periplasmic space [11]. However, the protein produced was insoluble indicating protein misfolding. In contrast, our N-terminally his_6_-thioredoxin tagged mature GSL1 fusion protein was found in the soluble cytoplasmic fraction when expressed in the same *E. coli* strain. Thioredoxin has previously been reported to be a useful partner for expressing cysteine-rich proteins [18]. As GSL peptides are highly cysteine-rich, this may also account for our successful production of soluble fusion protein. Whilst the GSL1 fusion protein was not expressed at the same high level as the thioredoxin fusion protein alone (Figure 2), milligram quantities of fusion protein could be IMAC purified in large culture volumes (Figure 3). This provided sufficient antigen for subsequent injection into rabbits to elicit a rabbit immune response and generate sera.

When it came to testing the potency of the anti-GSL1 sera it was not possible to use synthetically produced GSL1 peptide due to its lack of solubility. This is likely due to the structural nature of the peptides as GSL1 possesses 12 conserved cysteine residues [8, 9, 27] which would severely hamper the correct folding of a synthetically produced peptide. Instead we turned to a wheat germ cell-free expression system to generate N-terminally his_6_-tagged version of the GSL peptide as positive controls for western blot analysis. GSL1 was successfully translated in the wheat-germ cell free expression system and the anti-GSL1 serum was shown to selectively recognise the peptide (Figure 4). In addition, the GSL1 antibodies raised against protein that was expressed in the *E. coli* system were able to detect GSL1 peptides expressed in the plant-based wheat germ expression system. This highlights the robustness of using thioredoxin as a carrier molecule and the specificity of the antibodies raised against GSL1.

Some additional bands of higher molecular weight were detected by the anti-GSL1 serum in the wheat germ cell-free system in the western blot (Figure 4B). Because the western blot analysis was performed with total sera, and not purified antibodies, it is not surprising to see cross reactive bands, as the rabbit may have encountered wheat-based proteins in its diet to which it had produced antibodies. Most importantly, however, is our observation that only lane 2 (Figure 4B, arrow) corresponding to the wheat germ extract containing GSL1 peptide showed any reactivity of the correct size expected for the GSL1 peptide alone, while the control lane (1) was empty at the corresponding position.

We also used the DNA sequence for GSL2 [9] using the same strategy as described in this paper for the generation of soluble thioredoxin-GSL2 fusion protein, and immunisation of rabbits. However, we were unable to generate soluble GSL2 peptide using the same wheat-germ cell free expression system (data not shown). Commercially sourced GSL2 peptide was also not able to be solubilised in our hands. Therefore, with no positive control GSL2 peptide, we were unable to ascertain whether the sera obtained contained antibodies specific to GSL2. We were able to determine that sera generated from rabbit injected with his_6_-thioredoxin-GSL2 had no reactivity against the GSL1 peptide produced in the wheat-germ cell free expression system (data not shown). These observations highlight the difficulty in working with GSL peptides.

Although it was not attempted in our study, an additional advantage of our production of the soluble GSL1 thioredoxin fusion protein could be through using the thrombin and enterokinase protein cleavage sites present in the pET-32a vector. These cleavage sites lie between the end of the thioredoxin coding region and the GSL1 coding region. Separation of the GSL1 peptide from thioredoxin in the GSL1 fusion protein could be attempted by cleaving the fusion protein using thrombin and enterokinase enzymes. This could then be used to generate enough protein for structural studies by NMR or X-ray crystallography.

## Conclusions

We present here the first report of a robust method for the generation of GSL1 peptide expressed as a fusion protein with thioredoxin that has resulted in milligram quantities of soluble protein. We were able to demonstrate that a wheat germ cell-free system can be used to successfully express small quantities of GSL1 peptide useful as positive control in western blot analysis. The antibodies generated in rabbit after immunisation with the GSL1 fusion protein expressed in a prokaryotic bacterial system were able to recognise the GSL1 peptide produced in the eukaryotic wheat germ cell-free system. This further highlights the usefulness of thioredoxin as a fusion partner and the specificity of the antibodies we obtained. To our knowledge this is the first report of antibodies being produced against the GSL1 peptide.

## Methods

### Construction of recombinant expression vectors

The DNA coding sequence for the *GSL1* (Genbank accession FJ195646) gene from potato was used for this study. The DNA sequence encoding the GSL1 mature peptide was designed with *Eco*RI restriction sites (*GSL1*) at both the 5’ and 3’ends (Figure 1) to enable cloning into the pET-32a expression vector (Novagen, CN Biosciences, San Diego, CA, USA). The construct was *de novo* synthesised by Genscript Corporation (Piscataway, NJ, USA) and supplied in the plasmid pUC57 (designated pUC57-GSL1). Digestion of pUC57-GSL1 with *Eco*RI produced a DNA fragment of approximately 200 bp which was then ligated into the vector pET-32a using standard molecular biology techniques [28], resulting in the production of the *E. coli* expression vector pET-32a+GSL1. Positive clones with the *GSL1* gene in the correct orientation were identified by PCR screening using the sequencing primers GSL1 (5’GGGCATTTAGACTTGCCCTTA3’) combined with the S.Tag primer which binds just upstream of the multiple cloning site in pET-32a (5’CGAACGCCAGCACATGGACA3’).

To enable recombinant expression of an N-terminally his_6_-tagged GSL1 peptide in the wheat germ cell-free expression system, the *GSL1* gene was PCR amplified from the pUC57-GSL1 plasmid using the StSN1GWCHMP For primer (5’GGGGACAAGTTTGTACAAAAAAGCAGGCTATGCATCATCATCATCATCATGGTTCAAATTTTTGTGATTCAAAGTGCAAGC3’) and the StSN1GWCH Rev primer (5’GGGGACCACTTTGTACAAGAAAGCTGGGTTCAAGGGCATTTAGACTTGCCCTTAGAGTTC3’). The primers incorporate sequence encoding an N-terminal his_6_ tag, as well as attB1 and attB2 sequences to enable Gateway cloning of the fragment into pDONR221 using the BP clonase II enzyme (Life Technologies, Carlsbad, CA, USA). The construct was then transferred using the Gateway® system from this vector into pEU-DEST [26] for wheat germ expression, using the LR clonase II enzyme (Life Technologies) to create the expression vector pEU-his_6_-GSL1.

### Expression of his_6_-thioredoxin-GSL1 in *E. coli*

The plasmid pET-32a+GSL1 was transformed into the *E. coli* strain BL21 (DE3), which contains a T7 RNA polymerase gene for inducible protein expression [29]. A single transformed BL21 (DE3) colony was used to inoculate 50 mL LB broth containing 100 mg/L ampicillin and incubated overnight at 30°C. Two mL of the overnight culture were transferred into a flask containing fresh 50 mL LB medium with 100 mg/L ampicillin. The culture was grown with 200 rpm shaking at 37°C until the OD (600 nm) reached 0.8. The culture was induced by addition of 0.2 mL of 100 mM isopropyl-β-D-thiogalactopyranoside (IPTG). Immediately prior to induction, 1 mL of culture was removed as the zero time-point sample. One mL samples were removed 2 and 4 h after induction. Each 1 mL sample of cells was pelleted in a micro-centrifuge and the cells were washed with 1 mL of phosphate-buffered saline (PBS), pelleted, and stored at -20°C until further processing. For preparative-scale experiments, the remaining 47 mL of the overnight culture was then transferred into a flask containing 1250 mL of LB medium with 100 mg/L ampicillin, grown with 230 rpm at 37°C until an OD (600 nm) of 0.8 was reached. Expression of his_6_-thioredoxin-GSL1 was induced by the addition of 0.5 mM IPTG. Cells were harvested at 2 h post-induction.

### Purification of his_6_-thioredoxin-GSL1 fusion protein

Immobilised Metal Affinity Chromatography (IMAC) purification [30] was used with modifications to purify the his_6_-tagged fusion protein. The induced culture was processed by centrifugation at 4,500 × g for 20 min at 10°C. The cell pellet was weighed and resuspended by pipetting on ice in 2.2 mL/g pellet lysis buffer (0.1 M HEPES, 0.5 M NaCl, 10% glycerol, 0.01 M imidazole, supplemented with one tablet of Complete EDTA-free protease inhibitor (Roche Diagnostics, Auckland, New Zealand), and 2,000 U Benzonase per 100 mL buffer, pH 8.0) and stored at -80°C. Tubes containing 10 mL – 12.5 mL thawed and pooled cell suspension were placed in an ice bath and sonicated using a Branson Sonifier 250 (Branson, Danbury, CT, USA) on output setting five, for 1 min 15 s per cycle. A total of three cycles were carried out with a rest period of 2 min between cycles. The lysate was centrifuged at 20,000 × g for 30 min at 4°C, the supernatant was decanted and filtered through a 0.45 μm syringe filter (Pall Corporation, Cornwall, UK).

One mL HisTrap™ FF columns (GE Healthcare, Piscataway, NJ, USA) and a peristaltic pump were used to purify the fusion protein. Eluted fractions were monitored at A_280nm_ using an ISCO UA-5 Absorbance Monitor and collected with an ISCO Retriever II fraction collector (Isco, NE, USA). The flow-rate was set to 1 mL/min for each run. The column was equilibrated with 5 mL IMAC wash1 buffer (0.02 M HEPES, 0.5 M NaCl, 10% glycerol, 0.01 M imidazole, pH 7.5) and loaded with the lysate. The protein sample was loaded on the column, washed with 10 mL IMAC wash1 buffer followed by 6 mL IMAC wash2 buffer (0.02 M HEPES, 0.5 M NaCl, 10% glycerol, 0.025 M imidazole, pH 7.5). Bound protein was eluted from the column with 6 mL IMAC elution buffer (0.02 M HEPES, 0.5 M NaCl, 10% glycerol, 0.5 M imidazole, pH 7.5) as 0.5 mL aliquots. All steps were performed at 4°C.

After determining which fractions contained the fusion protein by SDS-PAGE, those fractions containing low amounts of protein were concentrated and desalted by Microcon® centrifugal filter (Millipore, MA, USA) with a molecular weight cut-off of 10 kDA. Following these procedures, all fractions were pooled and placed into pre-prepared dialysis tubing with a molecular weight cut-off of 12 kDa. Protein was concentrated by placing the dialysis tubing in a plastic tray and covering with granular sucrose before incubation at 4°C overnight. The protein in the tubing was subsequently equilibrated three times in 2 L PBS for 2 h. The first PBS dialysis buffer was supplemented with 125 mM imidazole to help prevent protein precipitation. After a third dialysis using PBS, the protein was transferred to 1.5 mL tubes and approximate total protein was determined by SDS-PAGE followed by verification of protein content by western blot analysis using anti-thioredoxin antibodies. The isolated fusion protein was used as an antigen for injection into rabbits, as previously described [31].

### Wheat germ cell-free expression of his_6_-tagged GSL1

Wheat germ cell-free expression was performed using the pEU-his_6_-GSL1 plasmid as template and the WEPRO7240H kit (CellFree Sciences, Yokohama, Japan) according to the manufacturers’ instructions. As this requires transcription and translation to be carried out separately, 20 μL transcription reactions were set up and incubated at 37 ^o^C for 6 h. Translation reactions were set up in individual wells of a 96-well microtiter plate using 10 μL of the transcription reaction mixed with 10 μL of the WEPRO mix and 0.8 μL of creatine kinase then overlaid with 206 μL of feeding solution. The reaction was incubated at 15°C for ~20 h before placing the samples on ice.

### SDS-PAGE and western blot analysis

SDS-PAGE analysis was performed essentially as described previously using a 10–20 % Tris-Tricine/Peptide Gel (Bio-Rad, Hercules, CA, USA) [25]. Protein was transferred onto the PVDF membrane using the iBlot gel transfer system (Life Technologies). For electrophoresis of protein produced in the wheat germ cell-free system, protein was electrophoresed using a Surelock Mini Cell (Life Technologies, Carlsbad, CA, USA), for 35 min at 200 V. Thioredoxin containing protein bands were visualised by incubation with the anti-thioredoxin primary antibody produced in rabbit (Sigma St. Louis, MO, USA), followed by the anti-rabbit-alkaline phosphatase secondary antibody (Sigma) and exposure to the chromogenic BCIP®/NBT-Blue liquid substrate (Sigma) (Figure 2B). GSL containing bands were visualised by incubation of the blot with a 1:400 dilution of sera containing Rabbit anti-GSL antibodies (raised in this study), followed by incubation with the secondary antibody Immun-Star™ Goat Anti-Rabbit (GAR)-HRP Conjugate (Bio-Rad) with a 1:100,000 dilution. Following the blots development, light signals were captured on Lumi-Film Chemiluminescent Detection Films (Bio-Rad) (Figure 4B).
